# Hepatitis E Virus ORF2 Protein Activates the Pro-Apoptotic Gene CHOP and Anti-Apoptotic Heat Shock Proteins

**DOI:** 10.1371/journal.pone.0025378

**Published:** 2011-09-23

**Authors:** Lijo John, Saijo Thomas, Ottmar Herchenröder, Brigitte M. Pützer, Stephan Schaefer

**Affiliations:** Department of Vectorology and Experimental Gene Therapy, Biomedical Research Center, University of Rostock Medical School, Rostock, Germany; University of Hong Kong, Hong Kong

## Abstract

**Background:**

Hepatitis E virus (HEV) is a non-enveloped plus-strand RNA virus that causes acute hepatitis. The capsid protein open reading frame 2 (ORF2) is known to induce endoplasmic reticulum stress in ORF2 expressing cells.

**Methodology/Principal Findings:**

In this study we found that HEV ORF2 activates the expression of the pro-apoptotic gene C/EBP homologous protein (CHOP). ORF2 stimulates the CHOP promoter mainly through AARE (amino acid response elements) and to a minor extent the ERSE (endoplasmic reticulum stress response elements). Activating transcription factor 4 (ATF4) protein binds and activates the AARE regulatory sites of the CHOP promoter. ORF2 expression also leads to increased phosphorylation of eukaryotic initiation factor 2 alpha (eIF2α) that in turn initiates the translation of ATF4 mRNA. The pro-apoptotic gene CHOP is an important trigger to initiate endoplasmic reticulum stress induced apoptosis. However, the activation of CHOP by ORF2 in this study did not induce apoptosis, nor did BCL2-associated X protein (Bax) translocate to mitochondria. Microarray analysis revealed an ORF2 specific increased expression of chaperones Hsp72, Hsp70B', and co-chaperone Hsp40. Co-immunoprecipitation (Co-IP) and *in silico* molecular docking analysis suggests that HEV ORF2 interacts with Hsp72. In addition, Hsp72 shows nuclear accumulation in ORF2 expressing cells.

**Conclusions/Significance:**

These data provide new insight into simultaneously occurring counter-acting effects of HEV ORF2 that may be part of a strategy to prevent host suicide before completion of the viral replication cycle.

## Introduction

Hepatitis E virus (HEV), the causative agent of viral hepatitis, is a non-enveloped positive-stranded RNA virus with an icosahedral capsid of about 27 to 34 nm in diameter [1], [2], [3]. The viral genome has three open reading frames called ORF1, ORF2, and ORF3. The nonstructural proteins required for virus replication and protein processing are encoded by ORF1, while ORF2 encodes the viral capsid protein, and ORF3 a small protein that regulates the cellular environment [4]. In insect cells, the truncated 56 kDa ORF2 protein can self-assemble to form virus-like particles which possess the same antigenic epitopes as the virion [5]. The N-terminal part of ORF2 is reported to contain an endoplasmic reticulum (ER) translocation signal [6], and its C-terminal region has an RNA binding site. ORF2's C-terminal region also contains several antigenic sites including a neutralization epitope ranging from residues 458 to 607 [7]. Studies investigating humoral responses against HEV showed prominent antibody responses against this and other linear or conformational epitopes of ORF2 [8], [9]. Thus, recombinant ORF2 protein has been used as a vaccine candidate [10], [11]. However, very little is known about the host cellular targets of ORF2 protein.

In eukaryotic cells, the ER is the primary site for post-translational modification, folding, and oligomerization of newly synthesized proteins [12]. Thus, many viruses including HEV exploit this cell organelle for their replication cycle. During the course of infection a large amount of viral proteins is synthesized in the cells and un- or misfolded proteins activate the ER stress response. The ER stress can lead to an activation of the unfolded protein response (UPR) which is mediated by three distinct branches namely inositol requiring enzyme 1 (IRE1), activating transcription factor (ATF6), and PKR-like ER kinase (PERK) [13]. Many viruses have developed distinct mechanisms to modulate these pathways [14], [15], [16]. Envelope proteins and replication of a hepatitis C virus replicon activate these pathways which lead to the induction of CHOP (also called GADD153) [17], [18]. However, different viruses use the three UPR branches differentially. While cytomegalovirus (CMV) favors the IRE1 branch and spare the ATF6 pathway [19], [20], the ORF3 protein of Severe Acute Respiratory Syndrome Corona virus (SARS Cov) promotes ER stress by activating the PERK pathway and CHOP [21]. Via its ER translocation signal HEV ORF2 enters the endoplasmic reticulum. However, a significant fraction of HEV ORF2 is present in the cytoplasm as a part of retro-translocation events [6]. The accumulation of ORF2 protein in the ER has been shown to activate the ER chaperones. The glucose-regulated proteins 78 kDa (GRP78) and 94 kDa (GRP94) are up-regulated in ORF2 expressing cells [6]. These chaperone proteins will refold the unfolded viral protein in an attempt to maintain homeostasis in the ER. If however, this adaptation fails, the apoptotic response is mediated mainly by ATF6 and ATF4 dependent activation of CHOP [22].

Heat shock proteins like Hsp72 (also called as HSPA1A) are well known for their anti-apoptotic properties. Amongst the heat shock family of proteins, Hsp72 is known to inhibit the stress induced c-Jun NH2-terminal kinase (JNK) signaling pathway [23], cytochrome release [24], and Bax translocation to mitochondria [25]. Nuclear accumulation of Hsp72 hinders apoptosis under stress conditions [26]. In this study we show that HEV ORF2 can activate CHOP by modulating the PERK eIF2α pathway. Microarray analysis shows that ORF2 protein up-regulates the chaperones Hsp72 and Hsp70B' as well as the co-chaperone Hsp40. Intriguingly, the activation of pro-apoptotic CHOP by ORF2 did neither lead to apoptosis, nor to the activation hallmarks of apoptosis signaling pathways like Bax translocation to mitochondria. Importantly, ORF2 protein interacts with Hsp72 and also increases its nuclear accumulation in ORF2 expressing cells. Hence the up-regulation of host survival mechanisms leading to nuclear accumulation of Hsp72 during ER stress induced by HEV ORF2 may be a viral evasion mechanism from cellular apoptosis.

## Results

### ORF2 activates the CHOP promoter

**Figure 1:**
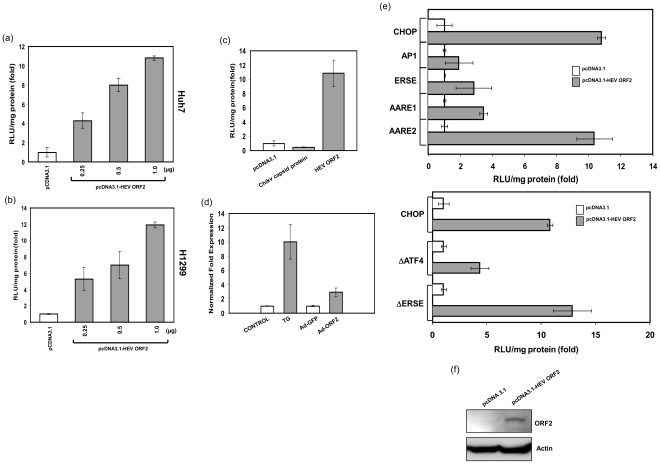
ORF2 transactivates the CHOP promoter in Huh7 and H1299 cells. (a) Huh7 cells were transfected with 100 ng of pGL3CHOP promoter luciferase reporter plasmid and pcDNA3.1-HEV ORF2 plasmid DNA as indicated. Cell extracts were prepared 48 hours after transfection and luciferase activity was determined. Data were normalized to total protein levels and are expressed as fold activation of pcDNA 3.1 alone (which was set as 1). (b) H1299 cells were transfected and analyzed as described in (a). (c) Huh7 cells were transfected with 0.1 µg of pGL3CHOP promoter luciferase reporter plasmid and 1.0 µg of pcDNA3.1-Chikungunya virus capsid. The samples were processed and analyzed as shown in (a). (d) Quantitative RT-PCR analysis of CHOP mRNA levels at 48 hpt with Ad-GFP or Ad-HEV ORF2. Cells treated with thapsigargin (TG, 4 µM) for 4 hours were used as positive control (e) The AARE1, AARE2, ERSE, AP1 enhancer luciferase reporter constructs of the CHOP promoter and promoter deletion constructs ΔATF4 and ΔERSE (0.1 µg each) were co-transfected in Huh7 cells along with 1.0 µg pcDNA3.1-HEV ORF2 plasmid. All data are representative of three independent experiments, SD are indicated by error bars. (f) ORF2 protein expression at 48 hours post-transfection was verified by Western blot analysis.

Processing of hepatitis E virus proteins leads to the accumulation in the ER of the viral capsid protein ORF2. The ER resident chaperones GRP78 and GRP94 as well as the protein disulfide isomerase have been shown to be up-regulated in ORF2 expressing cells [27]. A failure of this ER stress adaptation system and overexpression of the ER chaperones like GRP78 should also lead to the activation of pro-apoptotic downstream target genes like CHOP. Since ORF2 expression has been shown to induce ER stress and activation of ER chaperones, we analyzed whether expression of ORF2 had any effect on the expression of the pro-apoptotic gene CHOP. In a transient transfection system we looked at the transcriptional activation of CHOP in cells expressing the ORF2 protein. We have used luciferase reporter construct driven by the full-length 954 bp CHOP promoter and either pcDNA3.1 or pcDNA-HEV ORF2 was transiently transfected into hepatoma derived Huh7 and H1299 human lung cancer cells. We found that overexpression of ORF2 in Huh7 cells caused activation of the CHOP promoter in a dose-dependent manner (Fig. 1a). Similar findings were also observed in the non-hepatic cell line H1299 (Fig. 1b). As a control for the specificity of CHOP activation by HEV ORF2 we used the capsid protein of Chikungunya virus and found that it had no effect on CHOP promoter activity (Fig. 1c). The specificity of CHOP activation by ORF2 was also confirmed by the inability of ORF2 to activate a non-UPR gene promoter (data not shown). Moreover, we observed an increase of the CHOP mRNA levels in cells expressing ORF2 and in thapsigargin treated cells which served as a positive control compared to untreated cells (Fig. 1d).

### ORF2 activates both the AARE and ERSE elements of the CHOP promoter

The CHOP gene expression is regulated mainly through the regulatory sites ERSE, AARE1, and AARE2, respectively. In response to oxidative stress CHOP gene expression can also be activated through the AP1 element [28]. The response elements of the CHOP promoter are well characterized by promoter mapping studies and have been defined as follows: AARE2 (bases −778 to −770), AARE1 (bases −310 to −302), AP1 element (bases −244 to −238), and two ERSE (bases −103 to −76) [29], [30], [31], [32]. To investigate the relative contribution of these elements in response to HEV ORF2 protein expression, we employed different constructs with the isolated response elements (AARE1, AARE2, ERSE, AP1) of the CHOP promoter fused with the luciferase reporter. While AARE2 and AARE1 showed a 10- and 4-fold activation by HEV ORF2, the ERSE and AP1 elements of the CHOP promoter were only weakly activated by the capsid protein (Fig. 1e). We also tested the effect of promoter constructs with deletions encompassing the ERSE and activating transcription factor 4 (ATF4) binding sites of the CHOP promoter. Activation by HEV ORF2 was reduced upon the deletion of the ATF4 region as compared to the construct with deleted ERSE region of the CHOP promoter. These results suggest that ATF4 binding sites contribute to the major part of the CHOP promoter activation by the ORF2 protein. ORF2 protein expression was verified by Western blot (Fig. 1f).

### ORF2 activates the phosphorylation of eIF2α

**Figure 2:**
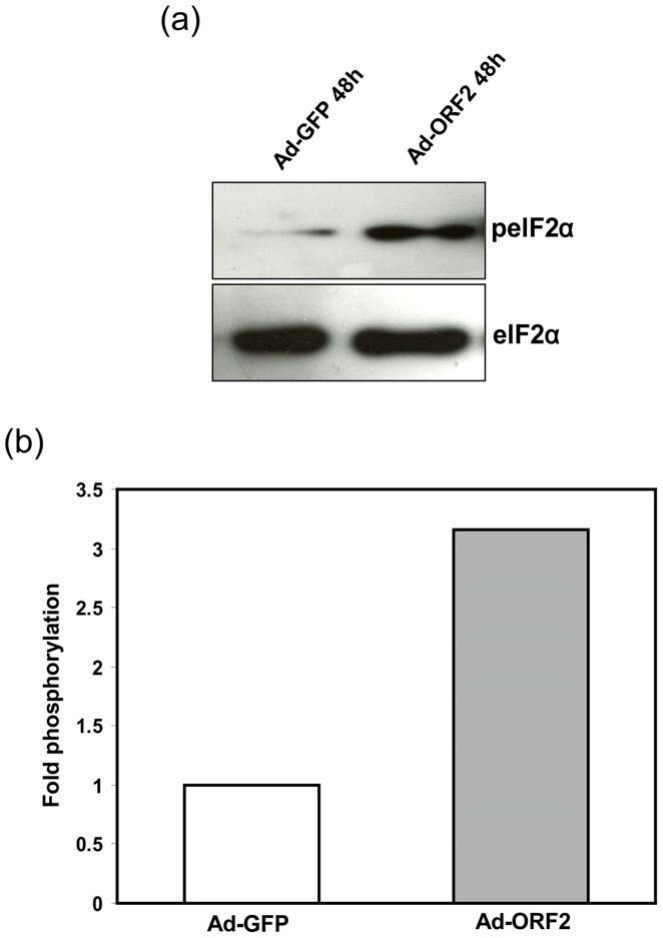
ORF2 activates PERK mediated eIF2α phosphorylation during ER stress. (a) Huh7 cells were transduced with Ad-GFP and Ad-ORF2 for 48 hours or treated with thapsigargin as a positive control for ER stress (2 µM) for 4 hours. Cell lysates were separated by SDS PAGE and analyzed for protein expression. (b) Signal intensities were quantified and phospho eIF2α signals were normalized to total eIF2α levels.

It is well established that ER stress leads to the activation of the three signaling branches of UPR [33]. Our results showed that ORF2 induced activation of the CHOP promoter was mediated mainly through the AARE regulatory sites. Transactivation of the AARE regulatory sites of the CHOP promoter is dependent on PERK mediated eIF2α phosphorylation and ATF4 translation [34], [35], [36]. We have also analyzed the phosphorylation status of eIF2α upon ORF2 expression. Fig. 2 (a) shows in cells transduced with Ad-ORF2 increased phosphorylation levels of eIF2α without a concomitant increase in the total eIF2α levels. Quantification of the eIF2α phosphorylation was threefold when compared with the control (Fig. 2b). These results suggest a possible mechanism for the transcriptional activation of AARE regulatory sites of the CHOP promoter by ORF2 through the phosphorylation of eIF2α.

### Activation of the pro-apoptotic gene CHOP by ORF2 does not induce Bax translocation to mitochondria

**Figure 3:**
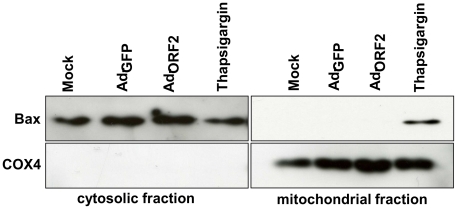
ORF2 does not induce Bax translocation from cytosol to mitochondria. Mitochondrial and cytosolic fractions were isolated from Huh7 cells infected with Ad-GFP and Ad-ORF2 for 72 hours, or treated with thapsigargin for 48 hours prior to harvest, and evaluated for Bax protein levels. The mitochondrial protein COX4 was used as control.

Induction of CHOP was reported to activate the ER stress induced major apoptotic pathways [37], [38]. Moreover, overexpression of CHOP leads to decrease in the B-cell CLL/lymphoma 2 (Bcl2) protein level and induces the translocation of Bax to mitochondria [39]. Under normal conditions Bax is located in the cytoplasm. During apoptotic events Bax undergoes conformational changes towards the pro-apoptotic state and is eventually translocated into mitochondria [40], [41]. So we investigated whether HEV ORF2 induced CHOP activation may lead to the translocation of Bax in Huh7 cells. At 72 hours post transduction (hpt) of Huh7 with either Ad-ORF2 or Ad-GFP, Bax quantitatively remained in the cytoplasm. In contrast, when the cells were treated with thapsigargin as a positive control, Bax was localized in the mitochondria (Fig. 3). Whereas thapsigargin efficiently induced apoptosis as detected by FACS analysis, no signs of apoptosis were found in ORF2 expressing cells (data not shown).

### Microarray analysis shows that ORF2 induces the up-regulation of Hsp70B', Hsp72, and Hsp40

**Figure 4:**
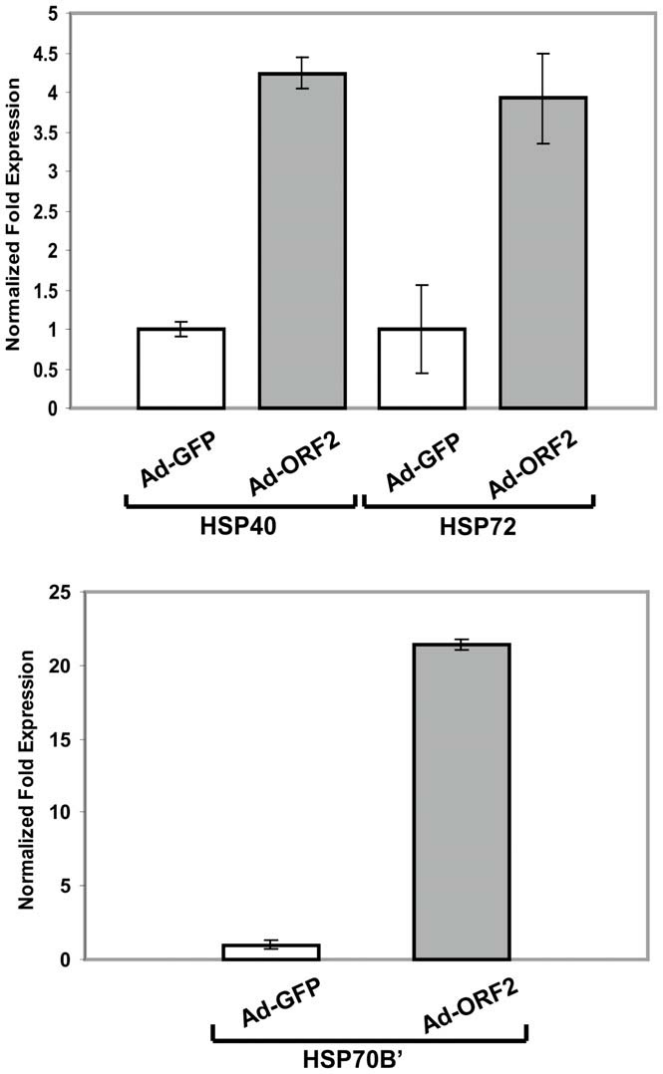
ORF2 up-regulates Hsp70B', Hsp72, and Hsp40. Quantitative RT-PCR analysis of heat shock proteins in Ad-ORF2 infected Huh7 cells. Expression levels were normalized to Ad-GFP transduced cells as control. Error bars indicate mean ± S.D. of three independent experiments.

The CHOP protein acts as a transcription factor and can differentially regulate the genes involved in either survival or death [42]. Overexpression of CHOP is known to regulate the protein levels of anti-apoptotic Bcl2 family proteins and the translocation of the pro-apoptotic protein Bax from the cytosol to mitochondria [37], [43]. Besides induction of CHOP, ORF2 may induce additional signaling pathways counteracting pro-apoptotic signals of CHOP. To unravel the consequences of the pro-apoptotic effects, we investigated the overall modulation of transcriptional changes induced by the expression of ORF2 protein. Microarray analysis of the Huh7 cell line transduced either with Ad-ORF2 or control Ad-GFP showed that expression of ORF2 induced a specific subset of chaperones like Hsp70B', Hsp72 as well as the co-chaperone Hsp40 (Table 1). qRT-PCR experiments confirmed the microarray results. ORF2 overexpression caused approximately 4-fold, 3-fold, and 20-fold up-regulation of Hsp40, Hsp72 (Fig. 4, upper panel), and Hsp70B' (lower panel), respectively. The heat shock family of proteins prevents the irreversible aggregation of unfolded proteins and keeps them competent for refolding [44], [45]. The microarray data suggest that the presence of ORF2 protein leads to the coordinated regulation of chaperones as well as the co-chaperone in response to protein damaging stress with an increased burden of non-native protein conformations.

**Table 1:** Chaperones and co-chaperones regulated by ORF2.

Affymetrix ID	Gene symbol	Description	Fold change
202581_at	Hsp72(HSPA1A)	Heat shock 70 kDa protein 1A	1.7
200800_s_at	Hsp72(HSPA1A)	-	1.9
1554334_a_at	Hsp40(DNAJ4)	DnaJ (Hsp40) homolog, subfamily A, member 4	2.2
117_at	Hsp70B'(HSPA6)	Heat shock 70 kDa protein 6 (Hsp70B')	3.5
213418_at	Hsp70B'(HSPA6)	-	7.6

The expression profiles of host genes significantly regulated (≥1.7 fold; *p* value≤0.05) at 60 hours post infection of Huh7 cells with Ad-ORF2 versus Ad-GFP using Affymetrix HGU_133 plus 2.0 array analysis. All values are results of three independent experiments and the fold changes were calculated as log 2 of signal log ratio using MAS5 (Microarray suite, Affymetrix).

### ORF2 interacts with Hsp72

**Figure 5:**
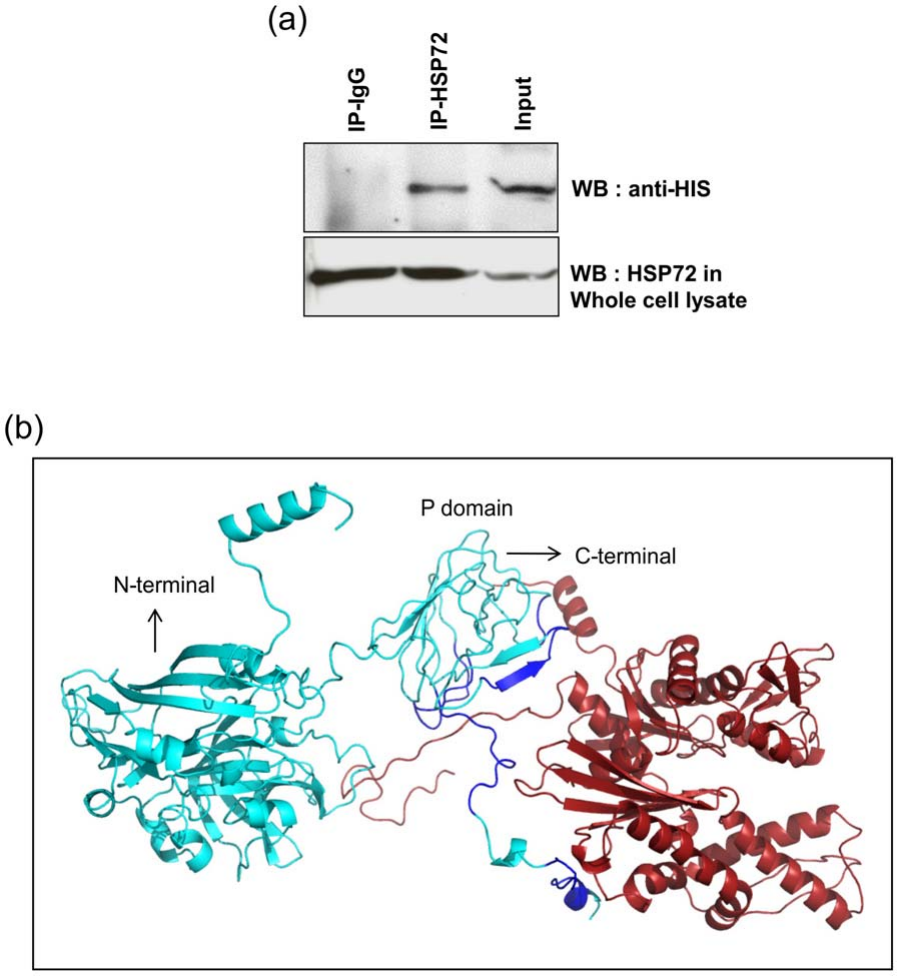
ORF2 interacts with Hsp72. (a) Cell lysates of HEK293 cells transfected with pcDNA3.1-HEV ORF2 were subjected to immunoprecipitation using anti-Hsp72 monoclonal antibody or control IgG. His-tagged ORF2 protein was detected from immunoprecipitates by Western blotting. (b) *In silico* modeling of ORF2 protein interactions with Hsp72. ORF2 and Hsp72 are displayed as a solid ribbon diagram, ORF2 (cyan), Hsp72 (firebrick red) and the interacting regions (blue). The N-terminal and C-terminal regions, and the P domain are indicated.

HEV ORF2 protein interacts with the members of the heat shock family of proteins GRP78 and Hsp90 [46], [47]. Since we observed increased expression of Hsp72 we also looked at a possible interaction of ORF2 with Hsp72. Experiments were performed to determine if ORF2 and Hsp72 directly interact in the cell. Protein extracts from transfected cells were subjected to Co-IP with an antibody against Hsp72 or control IgG, and subsequently analyzed through Western blotting with an anti-His-probe polyclonal antibody which detects His-tagged ORF2 protein. Representative results demonstrate that HEV ORF2 co-precipitated with Hsp72 and not with the control antibody (Fig. 5a). We also have used the *in silico* based docking analysis program to predict probable ways of interaction between Hsp72 and ORF2 protein (Fig. 5b, Supplementary Fig. S1). The best fit model scores a global energy (GE) value of −71.90, attractive and repulsive van der Waals force (Avwf) of −45.23, repulsive van der Waals energy (Rvdw), 21.10 and atomic contact energy (ACE)-17.66. The model indicates docking with the C-terminal or P domain of HEV ORF2 as shown before for its interaction with GRP78 [46].

### ORF2 induces the nuclear accumulation of Hsp72

**Figure 6:**
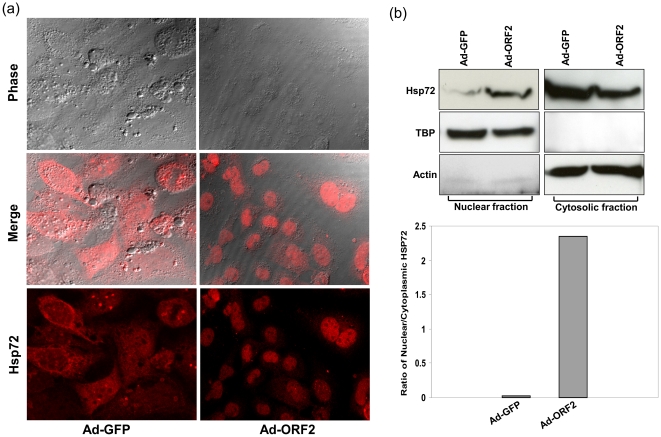
ORF2 increases nuclear accumulation of Hsp72. (a) Huh7 cells grown on cover slips were infected with Ad-GFP or Ad-ORF2, fixed and stained with anti-Hsp72 antibody at 72 hpt. Cells were imaged by confocal microscopy and composite images were created using IMAGE J software. (b) Western blot of Hsp72 protein expression in cytosolic and nuclear fractions of Ad-GFP and Ad-ORF2 infected Huh7 cells at 72 after transduction. Cytosolic actin and nuclear TBP were used for equal loading. Signal intensities of Hsp72 for both, nuclei and cytoplasm were quantified and normalized by appropriate loading controls. The nuclear/cytoplasmic ratio was calculated as described in methods.

The major heat shock protein Hsp72 is well known for its critical role in cell survival and its strong anti-apoptotic effects by modulating several pathways involved in apoptosis [48], [49]. As a part of its protective function, Hsp72 will migrate to the nucleus to execute extra chaperone activity in this compartment [45], [50]. To investigate ORF2 associated nuclear translocation of Hsp72, we checked for the nuclear accumulation in ORF2 expressing cells by immunofluorescence and cellular fractionation methods. Huh7 cells infected with Ad-ORF2 or Ad-GFP showed that ORF2 protein promotes nuclear accumulation of Hsp72 (Fig. 6a). This was further confirmed by cellular fractionation. Hsp72 was mostly detected in the nuclear fraction of ORF2 expressing cells (Fig. 6b).

## Discussion

The HEV ORF2 protein represents the capsid protein of hepatitis E virus. It is known to initially become accumulated in the ER and a fraction of this protein is translocated back to the cytoplasm. It also has an ER translocation signal for these retro-translocation events [27]. During the course of productive infection, the majority of viral proteins is synthesized by the host cell and processed through the cellular protein translocation machinery. Thus, the ER is an essential organelle for the replication and maturation of many viruses [51], [52]. Misfolded proteins will be retained in the ER until they reach native conformation or are translocated back to the cytosol to undergo a proteasome mediated degradation process [53]. HEV ORF2 protein was found to follow the retro-translocation pathway and remains in the cytosol without being a substrate of the 26s proteasome [27]. Overload with misfolded proteins in the ER will cause ER stress and eventually activate UPR pathways. ORF2 protein is known to induce ER stress and has been shown to activate the ER chaperones like GRP78 and GRP94 [27]. Up-regulation of these chaperones is associated with the UPR which binds to and retains the misfolded protein in the ER. ER stress response in mammalian cells involves the two major phases of adaptation and apoptosis. During the adaptation stage cells will use GRP78 and GRP94 to refold the unfolded proteins to maintain the ER homeostasis [54], [55]. If, however this adaptation process fails, then the pro-apoptotic process will be initiated by ATF6 and ATF4 dependent transcriptional activation of CHOP. Here we report that HEV ORF2 activates the full-length CHOP promoter and increases CHOP mRNA levels.

In mammalian cells ER stress is induced by three different pathways that are mediated by PKR-like ER kinase PERK, ATF6a and b as well as IRE1a and b [36]. Following ER stress IRE1 dimerizes and undergoes autophosphorylation following the activation of its endoribonuclease activity. Thus, activated IRE1a and IRE1b will cleave the substrate precursor XBP-1 mRNA to mature XBP-1 mRNA. The spliced form of XBP-1 has potential transcription activity and can bind to the ERSE element of the CHOP promoter. Indeed, during ER stress, activated PERK phosphorylates the subunit of eIF2α leading to a general attenuation of protein synthesis. This promotes translation of certain mRNAs such as the mRNA encoding the transcription factor ATF4 which also binds to the AARE regulatory sites of the CHOP promoter [56], [57]. Transcriptional regulation of the CHOP promoter is mainly triggered through the activation of the ERSE and AARE regulatory sites. We employed the promoter constructs with deletions of ERSE or transcription factor ATF4 binding region of CHOP. The deletion of ATF4 binding sites reduced the full promoter activity. In addition, we also observed a strong activation of AARE elements in ORF2 expressing cells. These results suggest that even though ORF2 protein induces the activation of the ERSE element, the transcription factor ATF4 binding regions like AARE1 and AARE2 play a major role in the activation of the CHOP promoter upon ORF2 expression. We have also analyzed the activation of the PERK pathway by ORF2 protein upon phosphorylation of eIF2α. We found that ORF2 increases the phosphorylation of eIF2α and thus confirmed the activation of the PERK-eIF2α pathway. Our results clearly show the specific activation of the pro-apoptotic gene CHOP and its responsive elements. Surprisingly, we did not find any indication of apoptosis in cells expressing HEV ORF2.

Activation of CHOP usually triggers the major apoptotic pathways and its overexpression will lead to down-regulation of Bcl2 protein levels and the translocation of Bax to mitochondria. The CHOP mediated death signals will be targeted to mitochondria, which may act as an integrator and amplifier of apoptotic pathways but the other mechanistic details for the direct involvement of CHOP are still unclear [43]. Bax is mainly located in the cytoplasm under non-apoptotic conditions and translocates to mitochondria in response to the apoptotic stimuli. Thus, Bax is a major player of the ER stress mediated apoptosis. In our studies, however, we have not found translocation of Bax to mitochondria as a consequence of ORF2 expression. This clearly suggests that activation of CHOP by ORF2 protein did not execute the apoptotic markers like Bax translocation events. These findings are in agreement with recent data of dengue virus infection. Dengue virus infection leads to CHOP activation but fails to induce any apoptotic markers like suppression of Bcl2 protein levels, activation of caspases or cleavage of poly (ADP-ribose) polymerase [58]. It has been reported that Hsp72 can inhibit CHOP and TNF-alpha induced apoptosis by binding to Bax and preventing its translocation to mitochondria [25], [39]. In agreement with this report we found that ORF2 expression up-regulates expression of Hsp72 and other chaperones. Expression levels of Hsp72 are rate limiting in control of ER stress and its overexpression helps cells to adapt to long-term ER stress *in vivo* by enhancing the IRE1alpha/XBP-1 branch of the UPR [59]. Co-IP experiments and *in silico* docking analysis revealed that ORF2 protein not only up-regulates, but also directly interacts with Hsp72.

Previous studies using molecular docking also showed that ORF2 interacts with chaperone GRP78 [46]. Our study showed similar results indicating that the docking occurs with the P domain of HEV ORF2. Thus the interaction of ORF2 with Hsp72 may be responsible to ensure correct protein conformation. This is in agreement with previous reports that HEV ORF2 protein is not a substrate of 26s proteasome complex and that the protease sensitive or ubiquitination sites of the protein were masked [27]. Induction of ER stress caused by ORF2 protein also induced nuclear accumulation of Hsp72. Overexpression of Hsp72 results in cytoplasmic localization and under conditions of stress such as heat shock it will translocate to the nucleus. Exposure to toxicants like dimethylarsinic acid also causes nuclear accumulation of Hsp72 and prevents apoptosis in human alveolar cells [26]. Nuclear accumulation of Hsp72 is mainly dependent on the phosphorylation status of tyrosine 524 and this nuclear translocation is important for cell survival [50]. Taken together, we clearly demonstrate that ORF2 induces the chaperones and a co-chaperone. This in turn may protect the host cells from ER stress mediated apoptosis during HEV infection. In summary, HEV ORF2 protein can activate the pro-apoptotic gene CHOP through its stress responsive elements in an ATF4 dependent manner. Furthermore, HEV ORF2 induced activation of CHOP leads to the up-regulation of chaperones like HS70B', Hsp72, and co-chaperone like Hsp40. In the light of our results we speculate that HEV ORF2 induced activation of Hsp72 and other chaperones may represent a survival mechanism in ORF2 expressing cells.

Altogether, our results explain the mechanism how a viral structural protein, although its expression can not avoid ER stress, does evade the consequences, namely the programmed cell death. The HEV capsid does so by exploiting the cell's chaperones and co-chaperones. Still, a robust infectious system for hepatitis E infection could ultimately verify this proposed mechanism of apoptosis delay by the whole virus. Finally, our studies will allow further investigation of the major heat shock protein like Hsp72 during HEV infection and could be exploited for therapeutic or diagnostic purposes.

## Materials and Methods

### Cells, plasmid constructs, and antibodies

H1299 and HEK293 cells were obtained from ATCC and Huh7 cells from R. Bartenschlager, University of Heidelberg, Germany. Cells were maintained in Dulbecco's modified Eagle's medium (DMEM) (PAA) supplemented with 10% fetal calf serum (Biochrom), 1% penicillin/streptomycin (PAA) and 0.5% amphotericin (PAA) in a humidified atmosphere of 5% CO_2_ at 37°C. HEV ORF2 was amplified with the forward primer (fwd) 5′-CCATGGGCATGCGCCCTCGGCCTATTTTG-3′, reverse primer (rev) 5′-CTCGAGTAACTCCCGAGTTTTACCCAC-3′ and the HEV clone pSK-HEV-2 (GenBank: AF444002.1), a kind gift of S. Emerson and R. Purcell (NIH, Maryland) [60] as a template. After amplification with PFU turbo polymerase (Stratagene), the amplimer was inserted into pcDNA™3.1D/V5-His-TOPO (Invitrogen). The pGL3CHOP-luciferase reporter plasmid containing the human CHOP promoter, the ERSE deletion constructs (−221 to −40) and ATF4 (−318 to −286) were obtained from P. Fafournoux, INRA, France [61]. The CHOP enhancer elements with luciferase reporter AARE1 (bases −310 to −302), AARE2 (bases −778 to −770), ERSE (bases −103 to −76), AP1 (bases −244 to −238) were kindly provided by S.C.M. Kwok, Albert Einstein Medical Center, USA [32]. The primary antibodies used were: mouse anti-Hsp72 (Stressgen Biotechnologies), rabbit anti-His probe, rabbit anti-Bax N-20 (Santa Cruz), rabbit anti-eIF2α, rabbit anti-phospho-eIF2α/Ser51, rabbit anti-COX IV (Cell Signaling Technology), mouse anti-TBP (Abcam), and mouse anti-β-Actin (Sigma). Secondary antibodies were: peroxidase-conjugated anti-mouse IgG, (GE Healthcare), anti-rabbit IgG (Cell Signaling Technology), and anti-mouse Alexa Fluor 633 (Molecular Probes). Cloning of the Chikungunya virus capsid protein into the pcDNA3.1 was performed using the construct described previously [62].

### Adenovirus construction

Adenovirus production utilized the pAdEasy-1 system of recombinant adenoviruses. The system uses homologous recombination in the recA+ Escherichia coli strain BJ5183 to introduce the gene of interest into the adenovirus background. Briefly, HEV ORF2 was amplified by PCR from the full-length HEV clone pSK-HEV-2 with primers fwd 5′-AGATCTATGCGCCCTCGGCCTATTTTG-3′, rev 5′-GTCGACAACTCCCGAGTTTTACCCACCTTC-3′, and cloned into the pAdTrack CMV vector using BglII and SalI restriction sites according to the methodology of the pAdEasy-1 system [63]. A multiplicity of infection of 20 was used for transduction.

### Microarray analysis

Gene expression microarray analysis was performed using Ad-ORF2 with Ad-GFP transduced Huh7 cells. Total RNA was isolated 60 hours post transduction using the RNeasy Mini Kit (Qiagen). Five micrograms of total RNA were used to prepare biotinylated cRNA probes which were hybridized to the Affymetrix Human Genome U133 Plus 2.0 Array according to the supplier's instructions (Affymetrix). Microarrays were analyzed by laser scanning (Affymetrix GeneChip Scanner 3000). Three independent experiments with two arrays per experiment (incorporating a dye swap), were undertaken and the data analyzed by the MAS5 (Microarray suite, Affymetrix). The fold changes were calculated as log 2 of signal log ratio with the cut-offs set at 1.7 fold and p-value cut-off at 0.05.

### Microarray data

All data were MIAME compliant and have been deposited in a respective database. The Gene expression omnibus (GEO) number of the microarray data associated with this paper is GSE29061.

### Quantitative Real-Time Polymerase Chain Reaction (qRT-PCR) analysis

RT-PCR was performed on total RNA prepared by Nucleospin RNAII (Macherey–Nagel). A total of 1 µg RNA was reverse transcribed using Omniscript RT (Qiagen) and Oligo-dT. The cDNA samples were mixed with Qiagen Quantitect Master Mix and run on a BIORAD iQ5 Multicolor Real-Time PCR Detection System using the primers as listed below. Hsp72 fwd: 5′-ACCTTCGACGTGTCCATCCTGA-3′ and rev 5′-TCCTCCACGAAGTGGTTCACCA-3′, Hsp70B' fwd: 5′-CCCTAAGGCTTTCCTCTTGC-3′ and rev 5′-CATGAAGCCGAGCAGTACAA-3′ and CHOP fwd: 5′-AGCTGGAACCTGAGG-3′ and rev 5′-TGGATCAGTCTGGAA3′. Expression levels of Hsp40/DNAJ4 were detected by using the specific TaqMan probe Hs00388055-m1α. Glyceraldehyde-3-phosphate dehydrogenase (GAPDH) was run as an endogenous control and all samples were normalized to the GAPDH expression levels.

### Western blotting and cellular fractionation

Cells were lysed in RIPA buffer (50 mM Tris-Cl, 150 mM NaCl, 1% NP-40, 0.5% sodium deoxycholate, 0.1% SDS) and the total protein concentration was quantified by the Bradford assay. Equal amounts of cellular protein were separated by sodiumdodecylsulfate polyacrylamide gel electrophoresis (SDS-PAGE) and immunoblotting was carried out as described previously [64]. Signal intensities of the bands were calculated using the TINA open source image analysis environment. For sub-cellular fractionation, the Apo Alert Cell Fractionation Kit (Clontech) was used according to the manufacturer's recommendations. Briefly, after 72 hpt cells were harvested, washed twice with PBS, resuspended in cell fractionation buffer and homogenized. Cytosolic and mitochondrial extracts were fractionated by differential centrifugation. Protein samples (50 µg) from both fractions were separated on 12% SDS-PAGE and detected with anti-COX IV and -Bax N-20 antibodies. The cytoplasmic and nuclear fractions and both protein samples were prepared 72 hpt using the Nuclear Extract kit (Active Motif). The cytoplasmic fractions were harvested as supernatants and the pellets were resuspended in 50 µl of complete lysis buffer and centrifuged at 14,000 g for 10 min at 4°C. Supernatants were collected as the nuclear fractions and both the protein samples were immunoblotted and probed with appropriate antibodies against Hsp72, TBP and β-Actin. Signal intensities of Hsp72 for both, nuclei and cytoplasm, were quantified and normalized to the appropriate loading controls and a nuclear cytoplasmic ratio of Hsp72 was calculated as described [50].

### Co-immunoprecipitation

A total of 200 µg of protein lysate from the HEK293 cells transfected with pcDNA3.1-HEV ORF2 were precipitated with 2 µg of Hsp72 antibody. Protein A/G agarose beads (Santa Cruz) were used to collect the immunoprecipitated complexes and the beads were washed with PBST before SDS-PAGE and Western blot analysis.

### Hsp72 immunofluorescence

Huh7 cells grown on coverslips were transduced with either Ad-GFP or Ad-ORF2. After 72 hours (h) cells were fixed with 4% paraformaldehyde in PBS and permeabilized with 0.2% Triton X-100. The coverslips were blocked with 5% BSA in PBS for 1 h at 37°C and incubated with anti-Hsp72 in a 1∶50 dilution as described elsewhere [65]. A secondary antibody conjugated to Alexa Fluor 633 was used for the visualization with a laser-scanning microscope.

### Luciferase reporter assay

Transfections were done in 6-cm plates (5×10^5^ cells per plate) using Effectene (Qiagen) and the cells were harvested after 48 h and lysed in cell lysis buffer (Promega). Luciferase activity was measured using the luciferase reporter assay system (Promega) and readings were taken on a luminometer. The readouts were normalized to the total protein concentration in the cell extract.

### Molecular docking analysis

The structures of Hsp72 and HEV ORF2 were modeled using the automated homology modeling server 3D-JIGSAW [66] which automatically selected the bovine structure of Hsc70 (PDB ID 1YUW) and PDB ID 2zzq for protein modeling. 3D-JIGSAW is an automated system to build three-dimensional models for proteins based on homologues of known structure. The template selection involves sequence alignments through database search for the best and fit model. PDB ID 1YUW has a sequence similarity of about 86% with the human HSPA1A (alias HSP72) making the model reliable. Crystal structures of HSPs of human origin having more than 70% sequence similarity include PDB ID 3LOF (Length: 113 amino acids) and PDB ID 3I33 (Length: 404 amino acids) compared to 554 amino acids for PDB ID 1YUW. Both PDB structures were submitted to Patchdock server [67] with Hsp72 to act as a receptor and ORF2 as a ligand with the default parameters. The top 1000 conformations were further refined using the Firedock server [68], [69]. Complexes with lowest global energy were selected and further analyzed. The figures were generated using pymol (http://www.pymol.org).

## Supporting Information

Figure S1 **Individual structures of ORF2 and HSP72.** (a) ORF2 is displayed as (cyan), and (b) Hsp72 (firebrick red) as a solid ribbon diagram.
(PPT)
