# Supplementary figures and images for: Hepatitis E Virus ORF2 Protein Activates the Pro-Apoptotic Gene CHOP and Anti-Apoptotic Heat Shock Proteins
Source: PLoS One. 2011 Sep 23;6(9):e25378. doi: 10.1371/journal.pone.0025378 (PMC3179511; doi:10.1371/journal.pone.0025378)

## Slide 1
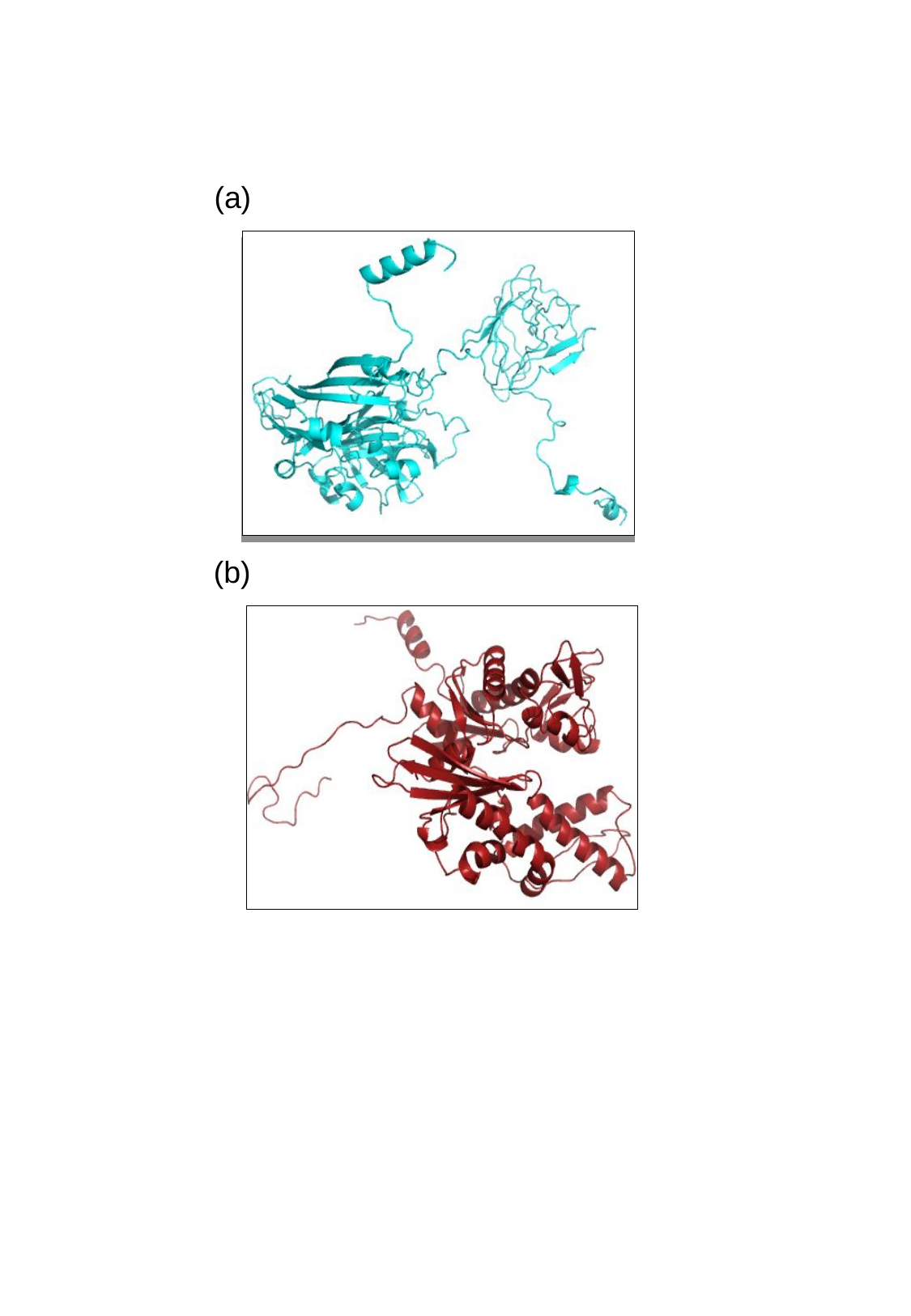

(a)
(b)

Supplement: Figure S1 — Individual structures of ORF2 and HSP72. (a) ORF2 is displayed as (cyan), and (b) Hsp72 (firebrick red) as a solid ribbon diagram. (PPT) [file pone.0025378.s001.ppt]
